# A Comparative Study of Cuff Pressure Changes of Endotracheal Tube with the Use of Air Versus Nitrous Oxide in the Anaesthetic Gas Mixture During Laparoscopic Surgery

**DOI:** 10.5152/TJAR.2022.21209

**Published:** 2022-08-01

**Authors:** Pradeesh Johny, Sivakumar Segaran, Mysore Venkatakrishnan Vidya, Mamie Zachariah, Sagiev George Koshy

**Affiliations:** Department of Anaesthesiology and Critical Care, Pondicherry Institute of Medical Sciences, India

**Keywords:** Air, endotracheal tube cuff pressure, laparoscopic surgeries, nitrous oxide, postoperative complications

## Abstract

**Objective:**

Cuffed endotracheal tube remains the standard of care during laparoscopic surgeries. Nitrous oxide is widely used as a carrier gas in anaesthesia practice. Cuff pressure greater than 30 cm H_2_O impairs tracheal mucosal perfusion leading to necrosis. This study aimed to compare the endotracheal tube cuff pressure while using air versus nitrous oxide in the anaesthetic gas mixture during laparoscopic surgeries.

**Methods:**

Sixty patients aged 18-70 years undergoing laparoscopic surgery were randomized into 2 groups of 30 each. Group 1 patients received air and oxygen in the anaesthetic gas mixture and group 2 patients received nitrous oxide and oxygen. Cuff pressure changes were measured throughout the surgery and perioperative hemodynamic parameters were recorded. The incidence and severity of postoperative sore throat were also evaluated postoperatively for 24 hours.

**Results:**

The increase in cuff pressure was more in the group using nitrous oxide (40.80 ± 8.78 cm H_2_O) when compared to the group using air (26.93 ± 2.69 cm H_2_O) (*P* < .001). Trendelenburg position was associated with a significant increase in cuff pressure when compared to other positions. The incidence and severity of postoperative sore throat were more at 2 hours (*P* = .0099) and 4 hours (*P* = .0105) postoperatively in the group using nitrous oxide. The hemodynamic parameters were comparable between the 2 groups.

**Conclusion:**

The use of nitrous oxide in the anaesthetic gas mixture causes an increase in endotracheal tube cuff pressure and increased severity of postoperative sore throat during laparoscopic surgeries.

## Introduction

Cuffed endotracheal tubes have become the standard of care for providing general anaesthesia because of the better seal of the trachea thereby preventing aspiration and theatre contamination with anaesthetic gases. The pressure inside the cuff should be good enough to prevent aspiration^1^ but not too high to obstruct tracheal blood flow.^2^ Cuff pressure greater than 30 cm H_2_O impairs tracheal mucosal perfusion (25 cm H_2_O)^2^ and can cause sore throat, hoarseness, dysphagia, and other complications.^3-6^

Nowadays, laparoscopic surgeries are gaining popularity because of a smaller incision, decreased pain, faster ambulation, and shorter hospital stay.^7^ For laparoscopic surgeries, general anaesthesia with cuffed endotracheal tube intubation and controlled ventilation remains the safe technique of anaesthesia. Balanced anaesthesia is provided by oxygen, volatile anaesthetics, and either nitrous oxide or air is used as a carrier gas, along with opioids and muscle relaxants. The use of nitrous oxide in general anaesthesia is advantageous taking into account its benefits like analgesia and minimizing the use of expensive volatile anaesthetics thereby reducing the cost and side effects of general anaesthesia and the prevention of intraoperative awareness.^8^

Laparoscopic surgeries usually cause an increase in endotracheal tube cuff pressure due to increased intra-abdominal pressure created by carbon dioxide insufflation.^9,10^ Endotracheal tube cuff pressure can also increase by the use of nitrous oxide, as it diffuses into the cuff.^11,12^ Endotracheal tube cuff pressures are not routinely measured during surgery and sometimes it becomes dangerously high causing tracheal mucosal necrosis.^13^

There is not much literature on the cuff pressure changes during laparoscopic surgeries while using nitrous oxide or air as a carrier gas, hence we aimed to compare the effect of air versus nitrous oxide on the endotracheal tube cuff pressure in patients undergoing laparoscopic surgery and to observe any postoperative complications.

## Methods

After obtaining institutional ethics committee approval (IEC-RC/18/65) and registering in the clinical trial registry (CTRI/2019/08/020558), this prospective randomized comparative trial was undertaken in a tertiary care hospital in Puducherry. After receiving the informed written consent, 60 patients who were scheduled for laparoscopic abdominal surgeries with American Society of Anesthesiologists Physical Status (ASA PS) I and II of either gender with 18-70 years of age were included in the study, and patients with a preoperative sore throat, upper respiratory tract infections, anticipated difficult airway, and previous oral surgeries were excluded from the study.

All 60 patients underwent pre-anaesthetic check-up the day before surgery and were kept nil per oral for 8 hours. They were premedicated with tab. metoclopramide 10 mg and tab. pantoprazole 40 mg the night before and on the day of surgery. On shifting to the operating room, all patients were connected to ASA standard monitors like electrocardiogram, pulse oximetry, capnography, and non-invasive blood pressure monitoring. Patients were randomized into 2 groups using computer-generated randomization. After preoxygenation with 100% oxygen for 3 minutes, general anaesthesia was induced with glycopyrrolate 5 µg kg^−1^ intravenously (i.v.), midazolam 30 µg kg^−1^ i.v., fentanyl 2 µg kg^−1^ i.v., propofol 2 mg kg^−1^ i.v., and endotracheal tube insertion was facilitated by vecuronium 0.1 mg kg^−1^ i.v. For all male patients, Portex endotracheal tubes of size 8-8.5 mm ID were used and for females, size 7-7.5 mm ID was used. The tube size was chosen based on the gender and stature (height) of the patient. After confirming bilateral air entry, the endotracheal tube’s cuff was inflated with air and cuff pressure was maintained at 25 cm H_2_O using a cuff pressure manometer (Smiths Medical ASD, Inc., Minneapolis, USA). Then mechanical ventilation was initiated and anaesthesia was maintained depending on the group allotted.

Group 1 received air in the anaesthetic gas mixture (air + O_2_ + isoflurane) and group 2 received nitrous oxide in the anaesthetic gas mixture (N_2_O + O_2_+ isoflurane). Endotracheal tube cuff pressure was measured subsequently every 30 minutes during the procedure using a cuff pressure manometer and tube position was checked with every change in patient position by auscultation. Whenever cuff pressure was found to be above 35 cm H_2_O at any point of time, air was removed from the cuff using a syringe to maintain a pressure of 25 cm H_2_O. The amount of air removed was quantified.

At the end of the procedure, the neuromuscular block was reversed with glycopyrrolate 10 µg kg^−1^ i.v. and neostigmine 50 µg kg^−1^ i.v. While extubating, gentle suction was done and patients were extubated. Patients who developed excessive bucking while extubation were excluded from the study. Then, the patients were shifted to the recovery room and provided with 100% oxygen and kept under observation. In the recovery room, 30 minutes after extubation, patients were assessed for sore throat, hoarseness, and dysphagia and thereafter at 2 hours, 4 hours, 6 hours, and 24 hours postoperatively, and sore throat was graded using the scale below:^14^

GRADE 0: No sore throat

GRADE 1: Mild sore throat (complains of sore throat only on asking)

GRADE 2: Moderate sore throat (complains of sore throat on his/her own)

GRADE 3: Severe sore throat (change of voice or hoarseness associated with dysphagia)

### Statistical Analysis

The sample size was calculated based on a study by Braz et al^15^ assuming the mean cuff pressure as 106 and standard deviation (SD) as 66 in the group using nitrous oxide and mean cuff pressure as 60.5 and SD as 38 in the group using air with a significance level of 5% and power of 80% the sample size required was 22 in each group. Considering 10% dropouts, the sample size was raised and rounded off to 30 in each group. Statistical Package for the Social Sciences (SPSS) 20.0 (IBM Corp.; Armonk, NY, USA) for Windows was used for statistical analysis and MS Excel was used for data management. All categorical data were expressed as numbers or percentages and measured using a chi-square test or Fischer’s exact test or analysis of variance. All numerical data were expressed as mean and SD and measured using an independent *t*-test. The amount of air removed was analysed using Mann–Whitney test. *P* <.05 was considered significant.

## Results

**Figure 1.**
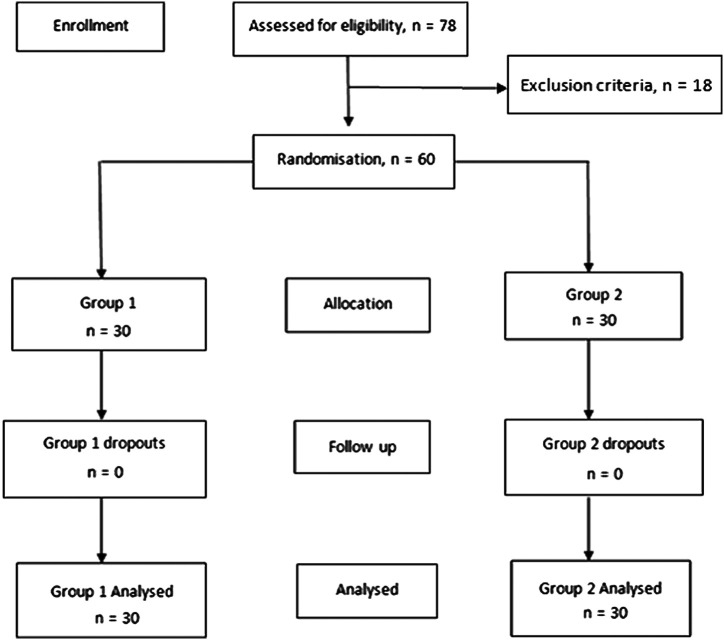
Consort diagram.

**Figure 2.**
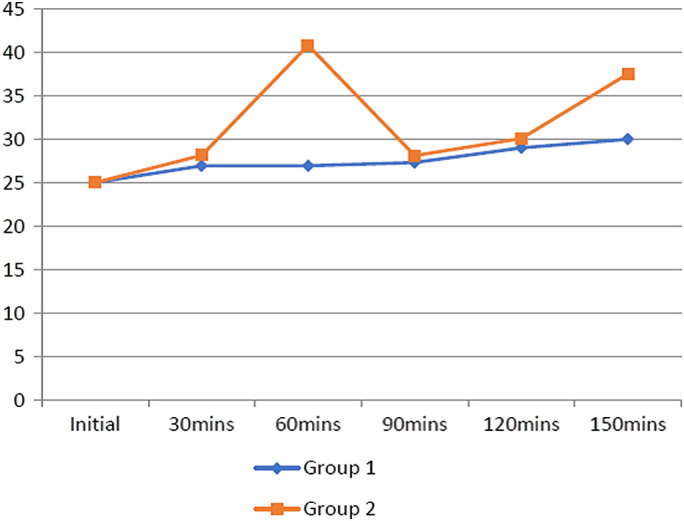
Cuff pressure at various time points among the 2 groups.

**Figure 3.**
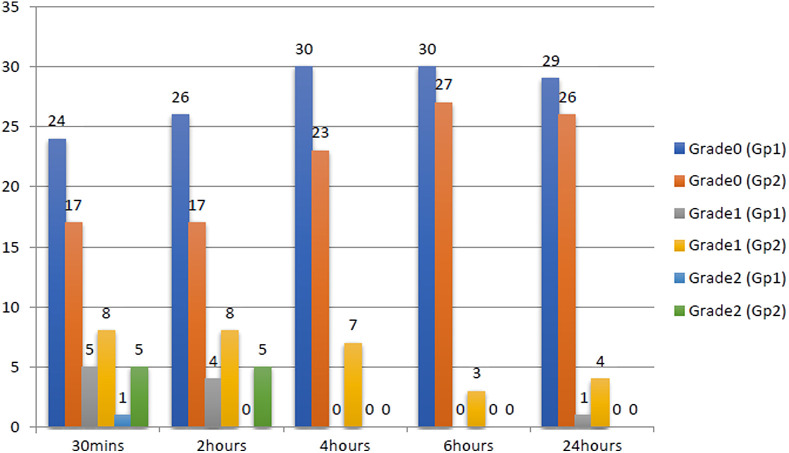
Distribution of severity of sore throat among the 2 groups at various time intervals.

In this study, 78 patients were assessed for eligibility out of which 18 patients were excluded because of exclusion criteria and 60 patients were randomized into 2 groups of 30 each. All 60 participants completed the study (Figure 1). The demographic data like age, gender, ASA grade, and body mass index were comparable between the groups and were not statistically significant (Table 1). Figure 2 shows the distribution of cuff pressures at different time intervals during the procedure in each group. At 60 minutes, the cuff pressure in group 2 was high (40 cm H_2_O) when compared to group 1 (26 cm H_2_O). In group 2, the cuff pressure gradually increased to 40.80 ± 8.78 cm H_2_O at 60 minutes and 26.93 ± 2.69 cm H_2_O at 90 minutes in group 1 and was statistically significant (Table 2). The cuff pressure changes in different positions at various time intervals in group 1 were not statistically different. In group 2, the cuff pressure in Trendelenburg position at 30 minutes and 60 minutes is significantly high when compared to neutral and reverse Trendelenburg position (Table 3). The incidence and severity of sore throat was higher in group 2 at second and fourth hour postoperatively when compared to group 1 and was statistically significant (Figure 3). No other complications were noted in both the groups.

## Discussion

In the present study, all 60 participants completed the study. We compared the cuff pressures during laparoscopic surgeries using nitrous oxide and air as anaesthetic gas mixture. We found that patients in the nitrous oxide group had higher cuff pressures (40 cm H_2_O) when compared to the air group (26 cm H_2_O). We also studied the effect of positioning on cuff pressure changes and found that the Trendelenburg position causes more cuff pressure changes than other positions in the nitrous oxide group. General anaesthesia has proven to be safe for laparoscopic surgeries as it allows precise control of ventilation. The cuff in the endotracheal tube is aimed to prevent gastric aspiration into the lungs and prevent leaks around the cuff thereby reducing theatre pollution. The recommended cuff pressure is between 20 cm and 30 cm H_2_O. An increase in endotracheal cuff pressure above 30 cm H_2_O causes compromise of tracheal mucosal blood flow and hence causes mucosal injury.^2^ Low cuff pressure may cause a risk of aspiration.^1^

Laparoscopic surgeries per se cause an increase in cuff pressure due to the creation of pneumoperitoneum which increases airway pressure thereby intracuff pressure.^16-18^ Nitrous oxide has higher blood solubility (0.46) than nitrogen (0.014) and so nitrous oxide moves faster into closed cavities than nitrogen. This leads to the expansion of gas-filled cavities.^19^ Nitrous oxide can also diffuse into air-filled endotracheal tube cuffs and thus cause an increase in cuff pressure^.8,10,20^

In this study, the cuff pressure started to increase in both the groups after 30 minutes, which can be explained by the pneumoperitoneum, the increase in cuff pressure in the nitrous oxide group was higher when compared to the air group at various time intervals and it was statistically significant at 60 minutes which may be due to diffusion of nitrous oxide into the cuff. This is similar to the study by Mogal et al^21^ where they compared the endotracheal cuff pressure changes during laparoscopic surgeries using air versus nitrous oxide in the anaesthetic mixture. They observed that cuff pressure in the group using nitrous oxide was higher when compared to the group using air.

To maintain the cuff pressure at 25 cm H_2_O, we had to remove the air from the cuff whenever it exceeded 35 cm H_2_O using a syringe. The amount of air removed from the cuff was quantified. In group 2, the cuff pressure exceeded > 40 cm H_2_O at 60 minutes; therefore, it was deflated to 25 cm H_2_O by aspiration of 1.7 mL of air. Subsequent measurements at 90 minutes and 120 minutes did not show any difference between the 2 groups. This may be because all the air in the cuff must have been replaced by nitrous oxide by this time.

Whenever the cuff pressures were found to be more than 35 cm H_2_O, the air was removed from the cuff which was quantified using a 2 mL syringe. In group 1, the cuff pressure never reached 35 cm H_2_O throughout the procedure.

The effect of patient positioning on cuff pressure during the procedure was also studied. The patients’ head was maintained in a neutral position throughout the procedure. Three positions namely neutral, Trendelenburg, and reverse Trendelenburg position were compared. In the Trendelenburg position, the cuff pressure was high and statistically significant when compared to the other positions.

Wu et al^17^ conducted a study on cuff pressure changes in laparoscopic surgeries in head-up and head-down positions. They concluded that the head-down position caused more increase in endotracheal cuff pressure than the head-up position which is similar to our study results. Brimacombe et al^22^ also conducted a study on different head positions causing changes in cuff pressure. They concluded that cuff pressure changes were minimal when the head was in neutral position.

We assessed for the incidence and severity of sore throat at 30 minutes, 2 hours, 4 hours, 6 hours, and 24 hours postoperatively. Postoperative complications were present in both the groups which were explained by laryngotracheal mucosal damage during intubation and patient positioning intraoperatively which causes an increase in cuff pressures. There was an increased incidence of sore throat among the group using nitrous oxide than in the group using air, which was statistically significant at 2 hours and 4 hours postoperatively. Lakhe et al^23^ conducted a study to evaluate cuff pressure and the incidence of postoperative sore throat. They observed an increase in cuff pressure and postoperative sore throat by using nitrous oxide in laparoscopic surgeries which is similar to our study results.

With regular cuff pressure monitoring and maintaining the cuff pressure in the recommended range, the incidence and severity of postoperative laryngotracheal complications can be significantly reduced. We found the presence of sore throat in both groups, but the incidence and severity of sore throat was very much reduced due to meticulous monitoring and cuff pressure adjustment.

## Conclusion

We conclude that when using nitrous oxide as anaesthetic gas mixture during laparoscopic surgeries, the magnitude of increase in intracuff pressure is very high when compared to air which contributed to more sore throat in the postoperative period. We also noticed that Trendelenburg position increases the intracuff pressure when compared to reverse Trendelenburg and neutral positions. We recommend the routine use of cuff pressure monitoring during laparoscopic procedures to decrease laryngotracheal morbidity.

## Figures and Tables

**Table 1.** Distribution of Demographic Data among the 2 Groups

Variable	Group 1	Group 2	*P*
Age (in years), mean ± SD	36.60 ± 11.19	40.13 ± 12.74	.258 (NS)
Gender n (%)			
Female	19 (63.33)	19 (63.33)	1.000 (NS)
Male	11 (36.66)	11 (36.66)
BMI (kg m^-2^), mean ± SD	23.13 ± 2.813	23.50 ± 2.502	.596 (NS)
ASA grade n (%)			
I	25 (83.33)	21 (70)	.222 (NS)
II	5 (16.66)	9 (30)
**Procedures**	N (%)	N (%)	Total
Diagnostic laparoscopy	6 (20)	4 (13.33)	10 (16.67)
Laparoscopic adhesiolysis	2 (6.67)	1 (3.33)	3 (5)
Laparoscopic appendicectomy	6 (20)	5 (16.67)	11 (18.33)
Laparoscopic cholecystectomy	13 (43.33)	18 (60)	31 (51.67)
Laparoscopic meshplasty	2 (6.67)	2 (6.67)	4 (6.67)
Laparoscopic ovarian cystectomy	1 (3.33)	0 (0)	1 (1.67)

NS, not significant; SD, standard deviation; BMI, body mass index.

**Table 2.** Distribution of Cuff Pressure (cm H_2_O) in the 2 Groups at Various Time Intervals

Time	Mean	Standard Error	95% CI		*P*
Lower Bound	Upper Bound
Group 1					
At intubation	25.000	0.000	25.000	25.000	.010 (S)
30 minutes	26.933	0.766	25.368	28.499	
60 minutes	26.933	0.491	25.929	27.938	
90 minutes	27.300	0.473	26.333	28.267
Group 2					
At intubation	25.000	0.000	25.000	25.000	<.001 (S)
30 minutes	28.133	0.849	26.396	29.870	
60 minutes	40.800	1.602	37.524	44.076	
90 minutes	28.100	0.796	26.473	29.727

**Table 3.** Distribution of Cuff Pressures in Various Patient Positions at Various Time Points in Group 2

Time Interval	Position	n	Cuff Pressure (cm H_2_O)		*P*
Mean	Standard D
At intubation	Neutral	7	25.00	0.000	
	Reverse Trendelenburg	18	25.00	0.000	
	Trendelenburg	5	25.00	0.000	
Total	30	25.00	0.000
30 minutes	Neutral	7	27.14	3.934	.022
	Reverse Trendelenburg	18	27.11	3.864	
	Trendelenburg	5	33.20	5.630	
Total	30	28.13	4.652
60 minutes	Neutral	7	38.00	7.416	.036
	Reverse Trendelenburg	18	33.60	10.479	
	Trendelenburg	5	43.89	7.584	
Total	30	40.80	8.775
90 minutes	Neutral	7	28.29	7.455	.342
	Reverse Trendelenburg	18	27.33	2.275	
	Trendelenburg	5	30.60	4.669	
Total	30	28.10	4.358
120 minutes	Neutral	3	31.67	11.547	.598
	Reverse Trendelenburg	9	29.56	3.005	
	Trendelenburg	0	.	.	
Total	12	30.08	5.632
150 minutes	Neutral	1	30.00	.	
	Reverse Trendelenburg	1	45.00	.	
	Trendelenburg	0	.	.	
Total	2	37.50	10.607
